# Structural transformation in Pd nanoclusters induced by Cu doping: an ADFT study

**DOI:** 10.1007/s00894-025-06305-y

**Published:** 2025-02-10

**Authors:** L. Santiago-Silva, H. Cruz-Martínez, H. Rojas-Chávez, L. López-Sosa, P. Calaminici

**Affiliations:** 1 Tecnológico Nacional de México, Instituto Tecnológico del Valle de Etla, Abasolo S/N, Barrio del Agua Buena, Santiago Suchilquitongo, 68230 Oaxaca, Mexico; 2 Tecnológico Nacional de México, Instituto Tecnológico de Tláhuac II, Camino Real 625, Jardines del Llano, 13550 San Juan Ixtayopan, Tláhuac, Ciudad de México , Mexico; 3 Departamento de Química, CINVESTAV, Instituto Politécnico Nacional 2508, 07360 San Pedro Zacatenco, Gustavo A. Madero, Ciudad de Mexico, Mexico

**Keywords:** ADFT, Nanoclusters, Chemical hardness, HOMO–LUMO gap

## Abstract

**Context:**

Transition metal nanoparticles have gained great importance due to their promising applications in various fields such as energy, electronics, medicine, and agriculture. For these applications, materials with outstanding properties are currently required. Therefore, different strategies have been established to improve the properties of pure nanoparticles such as alloying, doping, and formation of composites. Among these strategies, doping is gaining great importance because it has been demonstrated that doped nanoparticles have better properties than pure nanoparticles. Therefore, it is essential to know the role of doping on the structures and properties of clusters with more than 16 atoms. Consequently, in this study, we propose a theoretical study of structures and properties focusing on pure Pd_19_, Cu-doped Pd_18_ (Pd_18_Cu), and Cu_2_-doped Pd_17_ (Pd_17_Cu_2_) nanoclusters and thus elucidate the role of Cu atoms on the structures and properties of larger doped Pd nanoclusters than those already presented in the literature. We have selected a nanocluster with 19 atoms since the most stable structure of this system is characterized by defined shapes such as octahedron or double-icosahedron.

**Methods:**

Ground state structures and properties of Pd_19_, Pd_18_Cu, and Pd_17_Cu_2_ nanoclusters were studied using the auxiliary density functional theory (ADFT), as implemented in the deMon2k code. For obtaining the ground state structures of Pd_19_, Pd_18_Cu, and Pd_17_Cu_2_ nanoclusters, several dozen initial structures were taken along Born–Oppenheimer molecular dynamics (BOMD) trajectories and subsequently optimized without symmetry restrictions. The optimizations were performed with the revised PBE functional in combination with TZVP-GGA for the Cu atoms and using an 18-electron QECP|SD basis set for the Pd atoms. Different energetic and electronic properties were calculated for the most stable structures of Pd_19_, Pd_18_Cu, and Pd_17_Cu_2_ nanoclusters. Interestingly, when the Pd nanocluster is doped with two Cu atoms (Pd_17_Cu_2_), there is a structural transition, because the most stable structures for Pd_19_ and Pd_18_Cu are icosahedral. While the Pd_17_Cu nanocluster is characterized for a double-icosahedral-base structure. The binding energy per atom increases when the Cu concentration in the nanoclusters increases. According to the HOMO–LUMO gap, the chemical reactivity of the nanoclusters tends to increase as the Cu content in the nanoclusters increases.

**Supplementary Information:**

The online version contains supplementary material available at 10.1007/s00894-025-06305-y.

## Introduction

Transition metal nanoparticles have gained great importance due to their promising applications in various fields such as energy, electronics, medicine, and agriculture [1–10]. For these applications, materials with outstanding properties are currently required. Therefore, different strategies have been established to improve the properties of pure nanoparticles such as alloying, doping, and formation of composites [11–15]. Among these strategies, doping is gaining great importance because it has been demonstrated that doped nanoparticles have better properties than pure nanoparticles [16–19]. For instance, doped Pt_3_Ni nanocatalysts exhibited better activity and selectivity properties for the oxygen reduction reaction (ORR) than non-doped Pt_3_Ni catalysts [16]. Also, for Rh-doped PtNi octahedral nanoparticles, better results for ORR than non-doped PtNi octahedral nanoparticles are available [17]. In other studies, the same trends were found, where the catalytic activities of doped PtCu and PtCo nanoparticles for ORR were higher than those obtained for non-doped PtCu and PtCo nanoparticles [18, 19]. In addition, theoretical computations have demonstrated that doped metal clusters exhibit better properties than pure metal clusters [20–23]. These studies show the role that doping has for improving the properties of pure nanoparticles. Therefore, theoretical or experimental studies of doped nanoparticles are of great importance in various fields of knowledge.

Among the different nanoparticles studied to date, Pd-based nanoparticles have gained great attention for their outstanding applications in catalysis [24–27]. Consequently, the investigation of Pd-based nanoparticles is a very attractive topic for both experimental and theoretical areas. In this direction, various density functional theory (DFT) studies on doped Pd clusters have been performed [28–37]. For instance, structures and properties of Fe-doped Pd_n_ clusters (*n* = 1–13) were studied employing DFT calculations [28]. The Fe-doped Pd clusters presented higher binding energies and magnetic moments than pure Pd clusters. In another study, structures and properties of FePd_n_ clusters (*n* = 2–14) were studied using DFT calculations [29]. The results obtained were like those previously calculated [28]. Also, as the number of Pd atoms increased, the formation of core–shell structures was observed, where the Fe atom occupied the central position of the structure [28, 29]. Besides, the structures and properties of Mn-doped Pd_n_ clusters (*n* = 2–18) were investigated using DFT computations [30]. The bigger Mn-doped Pd clusters exhibited different structures than the pure Pd clusters. Also, the Mn-doped Pd clusters presented higher stabilities and magnetic moments than pure Pd clusters [30]. In another study, structures, relative stabilities, and magnetic properties of Al-doped Pd_n_ (*n* = 1–8) clusters were studied using DFT calculations [32]. The Al-doped Pd clusters presented lower magnetic moments than the pure Pd clusters. In another study, the structures and magnetic properties of Mo-doped Pd_n_ (*n* = 2–14) clusters were studied using DFT computations [35]. As the number of Pd atoms grows, the formation of core–shell-type structures is observed, being the Mo atom in the center of the structure. More recently, the structures and properties of Co-doped Pd_n_ (*n* = 1–12) clusters were investigated employing DFT calculations [36]. Co-doped Pd_n_ clusters exhibited higher stability and magnetic moments than pure Pd clusters. Finally, structures and energy properties of Ni- and Cu-doped Pd_n−1_ (*n* = 2–13) clusters were studied using auxiliary DFT (ADFT) calculations [37]. The doped Pd clusters presented better stabilities than the pure Pd clusters. Also, as the number of Pd atoms grows, the formation of core–shell structures was observed, with the Ni or Cu atom in the center of the structure. As the previous review shows, there are numerous theoretical studies available on doped Pd clusters. However, most of these studies are focused on clusters with less than 16 atoms [28, 29, 31–37]. Therefore, it is essential to know the role of doping on the structures and properties of clusters with more than 16 atoms. Consequently, in this study, we propose a theoretical study of structures and properties focusing on pure Pd_19_, Cu-doped Pd_18_ (Pd_18_Cu), and Cu_2_-doped Pd_17_ (Pd_17_Cu_2_) nanoclusters using ADFT calculations and thus elucidate the role of Cu atoms on the structures and properties of larger doped Pd nanoclusters than those already presented in the literature. In this work, a nanocluster with 19 atoms was selected, since generally, the lowest energy structure of this system is characterized by defined shapes such as double-icosahedron or octahedron. It is important to point out that this is the first study on this size and type of nanocluster. For the purpose of our theoretical investigation, we have employed the following working strategy. Born–Oppenheimer molecular dynamics (BOMD) simulations were used to better explore the potential energy surface of the nanoclusters of our interest. Along the BOMD trajectories, initial structures of these systems were extracted that were later optimized using the ADFT. After obtaining the most stable structures for these nanoclusters, we proceeded to calculate different energetic and electronic properties for these systems and thus the role of doping on the properties of Pd_19_, Pd_18_Cu, and Pd_17_Cu_2_ nanoclusters was determined.

The manuscript is organized as follows. The computational details are presented in the next section. In Section “Results and discussion,” the computed results are reported and discussed. In the last section, the conclusions are given.

## Computational details

All electronic structure computations were carried out using the ADFT methodology as implemented in the deMon2k code [38]. For the exchange and correlation contribution, the revised Perdew–Burke–Ernzerhof functional (rev-PBE) was employed [39]. The exchange–correlation potential was numerically integrated employing a fine grid. The variational fitting approach proposed by Dunlap and co-workers was used to compute the Coulomb energy [40]. The Pd atoms were treated using an 18-electron QECP|SD basis set [41], whereas the Cu atoms were treated considering a TZVP-GGA basis set [42]. The GEN-A2* auxiliary function set was employed for all calculations [42]. The computations were performed with the restricted open-shell Kohn–Sham (ROKS) methodology to avoid spin contamination [43]. It is important to highlight that the computational methodology used in this study has been validated in our previous studies [44, 45].

To determine the most stable structures of the Pd_19_, Pd_18_Cu, and Pd_17_Cu_2_ nanoclusters, more than 100 initial structures were optimized, which were extracted from BOMD trajectories. The BOMD trajectories were recorded at 2000 K with a total length of 20 ps. BOMD simulations for Pd_19_, Pd_18_Cu, and Pd_18_Cu_2_ nanoclusters were carried out with spin multiplicity of 9, 8, and 5, respectively. For all initial structures, a full structural optimization without any symmetry restriction was performed. A quasi-Newton optimization method in delocalized internal coordinates was employed in all calculations [46]. The most stable structures were characterized by numerical frequency analysis, which were obtained by diagonalizing the mass-weighted Cartesian force constant matrix. The spin density plots were made with the VUChem software [47].

## Results and discussion

### Most stable structures of Pd_19_, Pd_18_Cu, and Pd_17_Cu_2_ nanoclusters

**Fig. 1:**
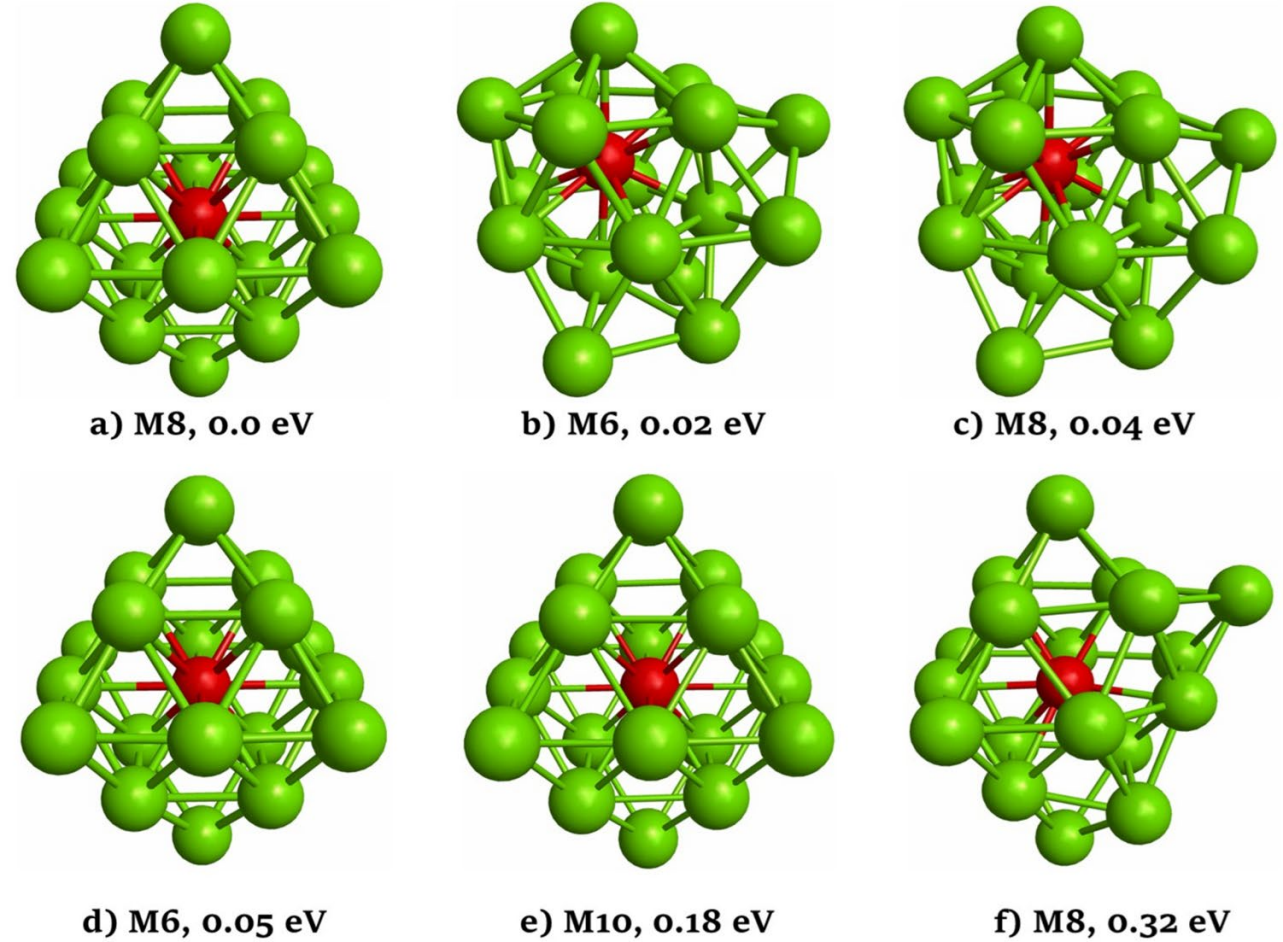
The most stable structures of the Pd_18_Cu nanocluster. Green and red spheres represent Pd and Cu atoms, respectively. The corresponding spin multiplicity (M) and the relative stability energy (in eV) of each cluster isomer with respect to the lowest energy structure are given

The most stable structure for the Pd_19_ nanocluster is an octahedral structure with a spin multiplicity of 9. This structure is like that one reported in the literature as the most stable structure for this type of nanocluster by several research groups [30, 48, 49]. Therefore, the obtained result indicates that the methodology used here is appropriate for the study of these nanoclusters. The six most stable structures for the Pd_18_Cu nanocluster are reported in Fig. 1. In Fig. 1, these stable isomers are labeled from (a) to (f), being (a) the lowest energy structure. Below each isomer structure, the corresponding spin multiplicity is indicated by the capital letter M, together with the relative energy, in eV, of each found stable isomer with respect to the most stable structure. As Fig. 1 shows, the most stable structure is octahedral and characterized by a spin multiplicity of 8. The isomer (b) has a distorted structure with a spin multiplicity of 6. The isomer (c) exhibits a structure very similar to structure (b) but with a spin multiplicity of 8. The isomer (d) possesses a structure similar to the one of isomer (a) but with a spin multiplicity of 6. Interestingly, we notice that the isomers (b) to (d) have energies very close to the most stable structure. Therefore, this shows that distinguishing the most stable structure for this type of system is a complicated task since several cluster isomers could be found within a very small energy window. The isomer (e) presents a structure like the most stable structure but with a higher spin multiplicity of 10, and it is located 0.18 eV above the most stable structure. Finally, isomer (f) has an octahedral base with a spin multiplicity of 8, and it is located 0.32 eV above the most stable structure. Because various of the isomers reported in Fig. 1 for the Pd_18_Cu nanocluster are isoenergetic, we have performed single-point energy calculations for all isomers reported in Fig. 1 employing the meta-GGA TPSS functional [50]. The relative stabilities calculated using this functional are reported in Fig. SI-1. When the rev-PBE functional was replaced by the TPSS functional in the singlepoint energy calculations, some changes in the relative energies were observed. For instance, isomers (a) and (b) exhibited the same energy. Also, the isomer (d) was more stable than the isomer (c) with the TPSS functional.

**Fig. 2:**
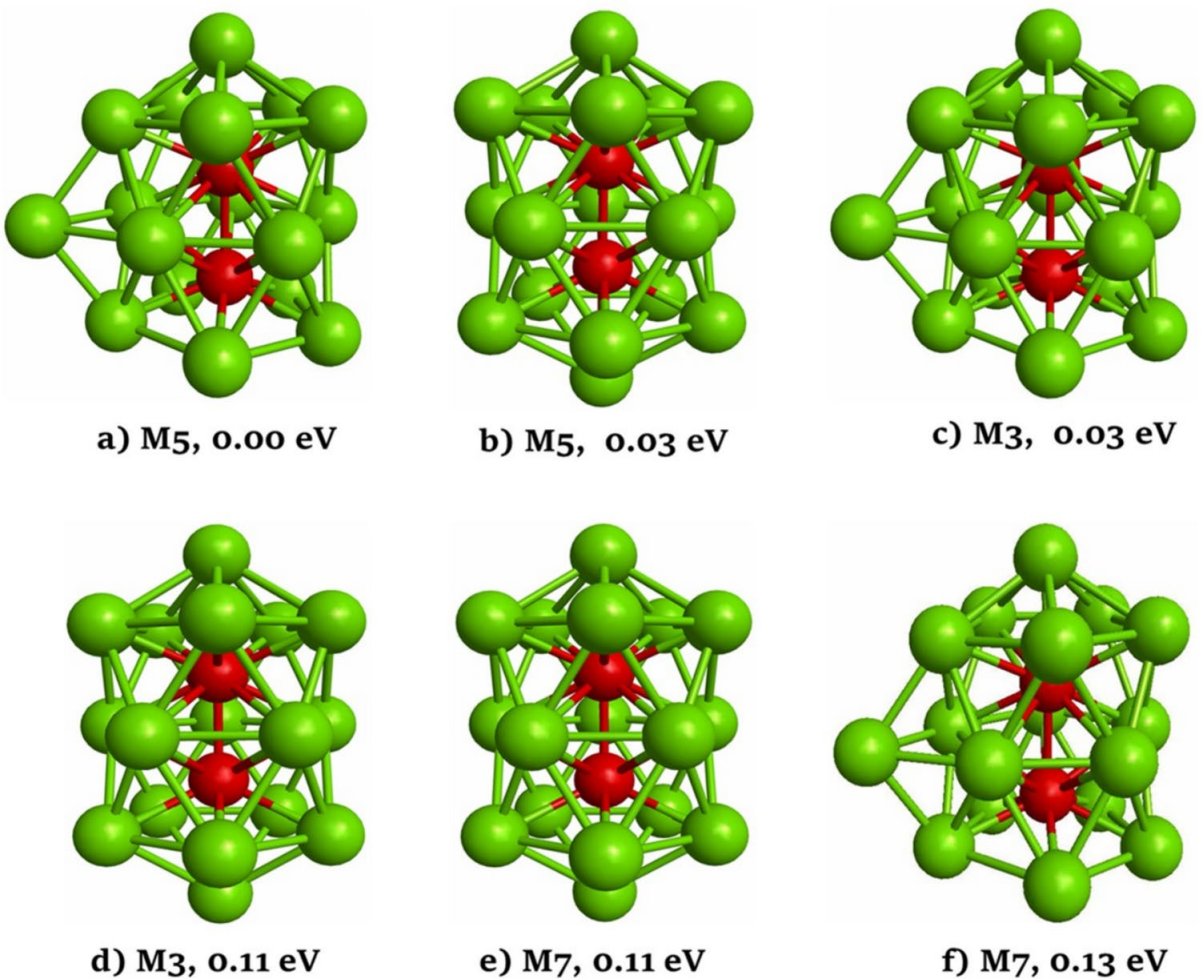
The most stable structures of the Pd_17_Cu_2_ nanoclusters. Green and red spheres represent Pd and Cu atoms, respectively. The corresponding spin multiplicity (M) and the relative stability energy (in eV) of each cluster isomer with respect to the lowest energy structure are given

The six most stable structures for the Pd_17_Cu_2_ nanocluster are graphically illustrated in Fig. 2. Turning to the analysis of the obtained results, we notice that, in this case, the most stable structure is an incomplete double-icosahedron with spin multiplicity of 5. The isomer (b) is a double-icosahedral with a spin multiplicity of 5. While the isomer (c) has an atom arrangement very similar to the one of isomer (a) but is characterized by triplet spin multiplicity. The isomers (b) and (c) exhibit energies very close to the most stable structure. The isomer (d) is like structure (b) but has a spin multiplicity of 3, and it is located 0.11 eV above the most stable structure. The isomer (e) is like the structures (b) and (d), and it is located 0.11 eV above the most stable structure. Finally, isomer (f) is like the structures of isomers (a) and (c) but with a spin multiplicity of 7, and it is located 0.13 eV higher in energy with respect to the most stable structure. Like the Pd_18_Cu nanocluster, for the Pd_17_Cu_2_ nanocluster, various of the isomers reported in Fig. 2 are isoenergetic. Therefore, we have performed single-point energy calculations for all isomers reported in Fig. 2 employing the meta-GGA TPSS functional. The relative stabilities calculated using this functional are reported in Fig. SI-2. When the rev-PBE functional was replaced by the TPSS functional in the single-point energy calculations, the relative stability energies showed slight changes. However, the order of the relative energies of isomers was not changed.

Interestingly, when the Pd nanocluster is doped with two Cu atoms (Pd_17_Cu_2_), we observe a structural transition because the Pd_17_Cu nanocluster is characterized by an incomplete double-icosahedral structure, while the most stable structures for Pd_19_ and Pd_18_Cu are octahedral.

To demonstrate that the investigated most stable structures for the Pd_19_, Pd_18_Cu, and Pd_17_Cu_2_ nanoclusters are minima, the numerical frequencies (in cm^−1^) of these structures were computed. The investigated harmonic frequencies for the most stable structures are real values (see Table 1), inferring the minimum nature of the investigated structures.

**Table 1:** The harmonic frequencies (cm^−1^) of the most stable structures for the Pd_19_, Pd_18_Cu, and Pd_17_Cu_2_ nanoclusters

Nanoclusters	Frequencies (in cm^−1^)									
Pd_19_	10	49	69	69	69	70	75	76	77	85
	88	89	91	92	95	96	98	98	101	102
	106	109	109	109	119	120	120	124	125	125
	145	150	150	152	153	154	155	171	171	172
	179	182	182	194	194	195	206	206	208	209
	212									
Pd_18_Cu_1_	34	35	62	64	65	66	66	67	77	77
	85	85	85	85	86	86	86	87	87	95
	96	99	100	101	110	110	111	130	130	131
	145	155	158	163	163	163	171	173	173	173
	184	184	185	200	201	201	220	220	225	225
	225									
Pd_17_Cu_2_	47	50	54	58	65	66	67	73	77	77
	80	84	84	85	89	89	95	100	102	103
	105	107	108	113	113	122	123	126	127	128
	128	130	136	137	142	161	164	164	170	171
	173	180	181	193	198	219	232	240	241	242
	247									

**Fig. 3:**
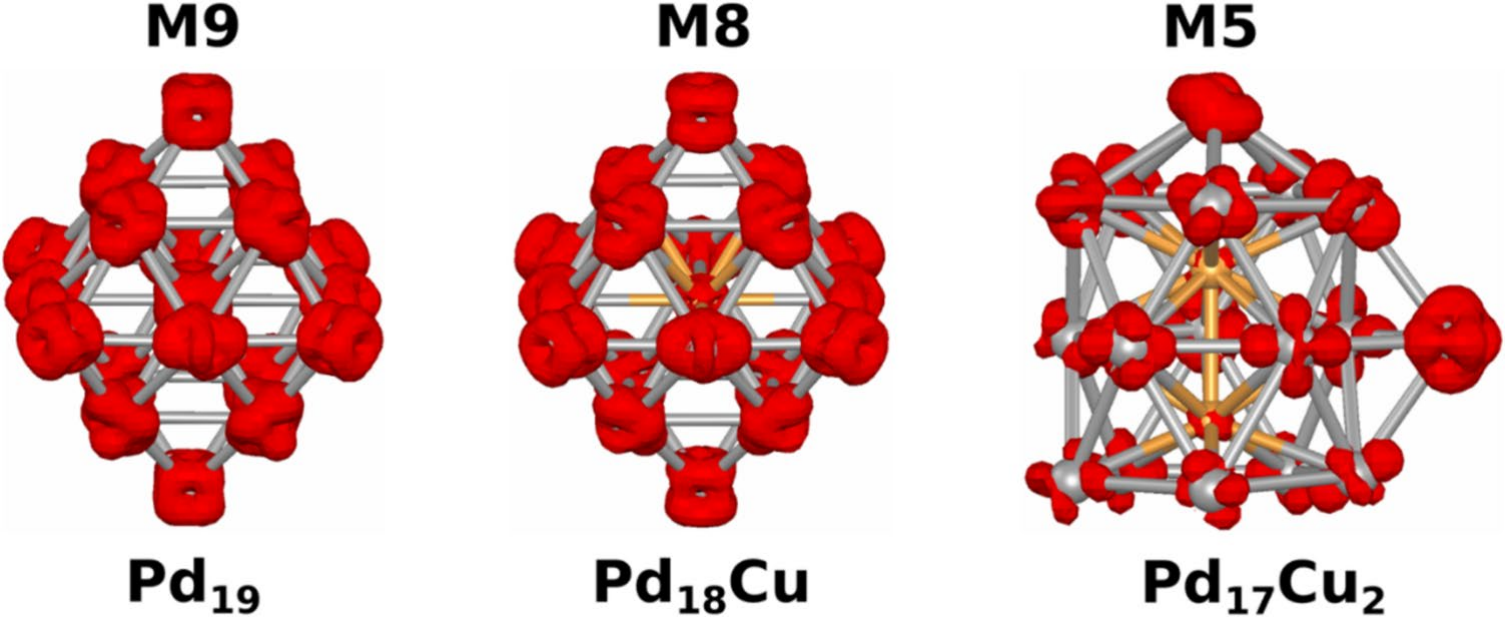
Spin density plots of the most stable structures of Pd_19_, Pd_18_Cu, and Pd_17_Cu_2_ nanoclusters

Turning to the analysis of the spin multiplicity of the most stable structures for the Pd_19_, Pd_18_Cu, and Pd_17_Cu_2_ nanoclusters, we can observe that the spin multiplicity tends to decrease when the concentration of Cu in the nanoclusters increases. This tendency can be associated with the electronic configuration of the Cu atoms alloyed to the Pd atoms since the Cu atoms are characterized by a closed shell configuration. To understand the sites where the spin densities are most allocated in these nanoclusters, spin densities of the most stable structures were plotted. The obtained results are presented in Fig. 3. For the Pd_19_ nanocluster, the spin density is distributed throughout the whole structure. For the Pd_18_Cu and Pd_17_Cu_2_ nanoclusters, the spin density is mainly distributed over the Pd atoms of the nanoclusters.

### Energetic, electronic, and magnetic properties of the Pd_19_, Pd_18_Cu, and Pd_17_Cu_2_ nanoclusters

To obtain insight into the stability and reactivity of the studied Pd_19_, Pd_18_Cu, and Pd_17_Cu_2_ nanoclusters, spin magnetic moment per atom (SMMA, in μ_B_), binding energy per atom (BEA, in eV), and chemical hardness (η, in eV) were calculated for the most stable lowest structures. It is observed that the SMMA tends to decrease when the Cu concentration increases in the nanoclusters (see Table 2). The BEA tends to increase when the Cu concentration in the nanoclusters increases. This result indicates that the stability of the nanoclusters increases due to the Cu atoms, which agrees with data reported in the literature for 3*d* transition metal doped palladium nanoclusters [28, 36, 37]. The η is similar for the three nanoclusters, suggesting a chemical reactivity closed of the doped nanoclusters with respect to pure palladium nanocluster.

**Table 2:** Spin magnetic moment per atom (in μ_B_), binding energy per atom (in eV), and hardness (in eV) of the most stable structures for the Pd_19_, Pd_18_Cu, and Pd_17_Cu_2_ nanoclusters

Nanoclusters	Spin magnetic moment per atom (μ_B_)	Binding energy per atom (eV)	Hardness (eV)
Pd_19_	0.42	2.29	1.64
Pd_18_Cu	0.37	2.32	1.65
Pd17Cu_2_	0.21	2.37	1.64

**Fig. 4:**
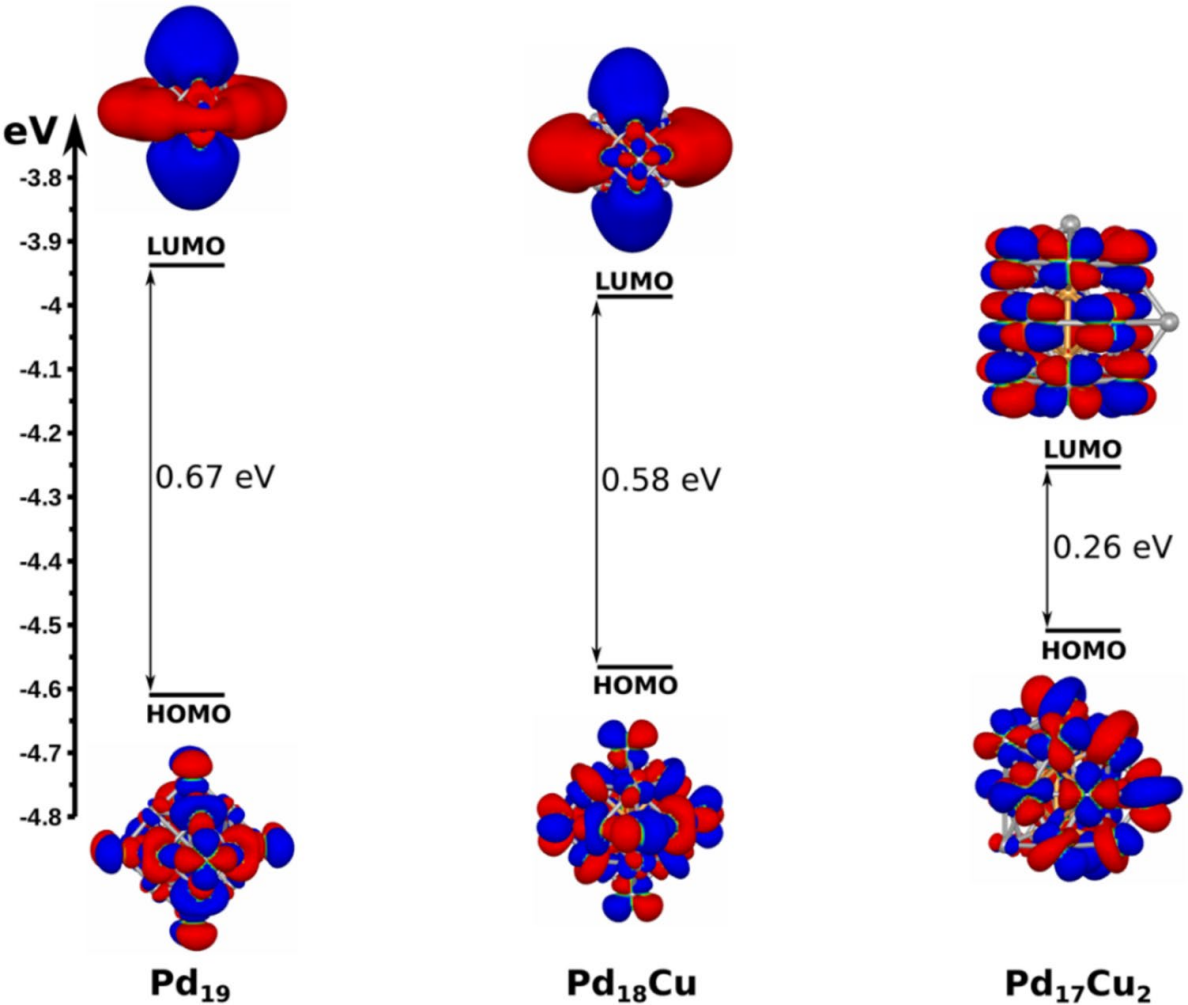
Highest occupied molecular orbital (HOMO) and lowest unoccupied molecular orbital (LUMO) plots and their energy gap for Pd_19_, Pd_18_Cu, and Pd_17_Cu_2_ nanoclusters

To gain insight into the chemical reactivity of these nanoclusters, the energy differences between the highest occupied molecular orbital and lowest unoccupied molecular orbital (HOMO–LUMO Gap) were calculated, too. The obtained results are presented in Fig. 4. It is observed that as the Cu concentration increases in the Cu-doped Pd nanoclusters, the HOMO–LUMO Gap tends to decrease, which suggests an improvement in the chemical reactivity of this type of system.

## Conclusions

Ground state structures and properties of Pd_19_, Pd_18_Cu, and Pd_17_Cu_2_ nanoclusters were studied employing the ADFT. This is the first ADFT-based theoretical study on the structures and properties of 3*d* transition metal-doped Pd nanoclusters with 19 atoms. It was observed that when the palladium nanocluster is doped with two Cu atoms (Pd_17_Cu_2_), a structural transition occurs because the most stable structures for Pd_19_ and Pd_18_Cu are octahedral, while the Pd_17_Cu_2_ nanocluster is characterized for an incomplete double-icosahedral structure. It is observed that the SMMA tends to decrease when the Cu concentration increases in these nanoclusters. On the other hand, the BEA tends to increase when the Cu concentration in the nanoclusters increases, inferring that the stability of the nanoclusters increases due to the Cu atoms. Based on the calculated HOMO–LUMO Gap, the chemical reactivity of these nanoclusters tends to increase as the Cu content in the nanoclusters increases.

## Supplementary Information

Below is the link to the electronic supplementary material.

Supplementary file1 Cartesian coordinates of the obtained most stable structures for the Pd18Cu and Pd17Cu2 nanoclusters employing the rev-PBE functional in combination with the QECP18-SD basis set for the Pd atoms and the TZVP-GGA basis set for the Cu atoms are provided in Supplementary Information (PDF 290 KB)

## Data Availability

No datasets were generated or analysed during the current study.
