# Supplementary material for: Structural transformation in Pd nanoclusters induced by Cu doping: an ADFT study
Source: J Mol Model. 2025 Feb 10;31(3):79. doi: 10.1007/s00894-025-06305-y (PMC11811464; doi:10.1007/s00894-025-06305-y)
Supplement: Supplementary file 1 — Supplementary file1 Cartesian coordinates of the obtained most stable structures for the Pd18Cu and Pd17Cu2 nanoclusters employing the rev-PBE functional in combination with the QECP18-SD basis set for the Pd atoms and the TZVP-GGA basis set for the Cu atoms are provided in Supplementary Information (PDF 290 KB) [file 894_2025_6305_MOESM1_ESM.pdf]

Structural transformation in Pd nanoclusters induced by Cu doping:

An ADFT study

L. Santiago-Silva[1], H. Cruz-Martínez[1, *], H. Rojas-Chávez[2], L. López-Sosa[3],

P. Calaminici[3, *]

[1]Tecnológico Nacional de México, Instituto Tecnológico del Valle de Etla, Abasolo S/N, Barrio del Agua Buena, Santiago Suchilquitongo, Oaxaca 68230, Mexico

[2]Tecnológico Nacional de México, Instituto Tecnológico de Tláhuac II, Camino Real 625, Jardines del Llano, San Juan Ixtayopan, Tláhuac, Ciudad de México 13550, Mexico

[3]Departamento de Química, CINVESTAV, Instituto Politécnico Nacional 2508, San Pedro Zacatenco, Gustavo A. Madero, Ciudad de Mexico 07360, Mexico

1.- More stable structures for the $Pd_{18}Cu$ nanocluster

Isomer a)

CU	-0.003326	-0.002513	-0.001011
PD	-0.219167	-2.174419	-1.686736
PD	1.804560	-0.338463	-2.060117
PD	3.740953	-0.184265	-0.246920
PD	2.082581	0.143816	1.798091
PD	0.274319	0.470576	3.708723
PD	0.060365	-1.693961	2.182390
PD	-2.022794	-1.838651	0.378145
PD	-2.089726	-0.146773	-1.798429
PD	-0.274409	-0.461602	-3.708345
PD	-0.066176	1.687003	-2.173287
PD	-1.871408	2.034142	-0.118050
PD	-3.735358	0.185132	0.257415
PD	-1.799592	0.338731	2.061428
PD	0.222013	2.175496	1.676290
PD	2.028375	1.835836	-0.380727
PD	0.154951	3.712603	-0.481097
PD	1.865410	-2.029529	0.118221
PD	-0.152912	-3.714171	0.473609

Isomer b)

PD	1.655060	-1.079763	0.242624
CU	-0.845027	-0.306947	0.116408
PD	0.899436	1.317330	-1.083585
PD	-0.325308	-2.913114	-0.171379
PD	-2.383218	-1.637113	-1.551323
PD	0.649925	1.021414	1.848358
PD	-0.025373	-1.589065	2.314874
PD	-1.937575	0.423253	2.421571
PD	0.208730	-1.051727	-2.212582
PD	1.025415	3.360157	0.561928
PD	1.998132	-2.945792	-1.617259
PD	-1.693421	0.951908	-2.049081
PD	2.796120	-0.327501	-2.035784
PD	-1.423431	2.221817	0.457354
PD	3.015578	1.265227	0.601506
PD	-2.524633	-2.015306	1.193496
PD	-3.410739	0.392342	0.052613
PD	-0.554994	3.421200	-1.738324
PD	2.534882	-0.631982	2.695482

Isomer c)

PD	1.399933	-1.374785	0.279031
CU	-0.903294	-0.141731	0.083671
PD	1.177093	1.106835	-1.074810
PD	-0.882470	-2.791619	-0.215480
PD	-2.614961	-1.125892	-1.643198
PD	0.753334	0.885781	1.858541
PD	-0.419453	-1.543418	2.304078
PD	-1.937362	0.748956	2.361379
PD	0.077055	-1.080011	-2.216934
PD	1.716962	3.075714	0.579424
PD	1.455490	-3.278828	-1.574316
PD	-1.380143	1.296131	-2.061050
PD	2.762726	-0.893188	-1.951526
PD	-0.982311	2.454734	0.429541
PD	3.162011	0.701202	0.717109
PD	-2.920159	-1.510650	1.099830
PD	-3.298585	1.018829	-0.023226
PD	0.216508	3.509102	-1.688574
PD	2.253710	-1.114263	2.770217

Isomer d)

CU	0.000583	-0.000568	0.001518
PD	0.984043	-2.554615	0.436988
PD	2.730166	-0.489839	-0.189787
PD	3.064351	1.447959	1.562216
PD	0.491175	2.011434	1.856503
PD	-2.131333	2.383599	1.921360
PD	-1.250376	-0.053854	2.484877
PD	-1.738943	-2.065140	0.629302
PD	-0.494622	-2.014250	-1.857597
PD	2.131700	-2.381022	-1.924439
PD	1.245812	0.052386	-2.479648
PD	-1.477903	0.543450	-2.295111
PD	-3.066990	-1.446245	-1.557913
PD	-2.727025	0.491096	0.190744
PD	-0.981148	2.553691	-0.440604
PD	1.742027	2.066098	-0.627932
PD	0.257808	2.492752	-2.757291
PD	1.474891	-0.543085	2.292063
PD	-0.253984	-2.494074	2.755361

Isomer e)

CU	0.035615	0.001214	-0.004736
PD	0.028599	-1.818728	-2.001549
PD	1.983609	0.109116	-1.959875
PD	3.782211	0.018488	0.003058
PD	1.981510	-0.087169	1.963814
PD	-0.019777	-0.183014	3.730239
PD	0.022196	-1.999551	1.815606
PD	-1.975006	-1.944761	-0.096750
PD	-1.973760	0.079335	-1.923535
PD	-0.012499	0.182304	-3.723531
PD	0.002535	2.007812	-1.814983
PD	-1.996999	1.927658	0.090962
PD	-3.824874	-0.019765	-0.004681
PD	-1.975031	-0.102864	1.918915
PD	0.006591	1.811728	2.001041
PD	1.979941	1.982165	0.093220
PD	-0.035291	3.734043	0.186582
PD	2.002998	-1.964725	-0.095479
PD	0.001781	-3.732798	-0.180224

Isomer f)

PD	1.279690	2.150273	-1.332791
PD	1.154936	-2.531583	-1.790334
PD	3.458104	1.811562	0.216933
PD	0.660310	-2.221379	0.854455
PD	2.398651	-0.295657	1.630425
PD	-1.225315	2.455981	-0.126249
PD	-1.283409	2.269249	2.550954
PD	-0.174223	-0.178020	2.567915
PD	-3.788765	-1.482255	-0.186284
PD	3.217854	-2.480935	0.212424
PD	-2.510978	0.356264	1.189652
PD	-2.432530	0.399886	-1.579314
PD	2.326443	-0.294079	-0.976107
CU	-0.125083	0.124626	-0.002032
PD	0.093382	-0.160927	-2.567527
PD	-0.959572	2.263111	-2.763403
PD	1.094759	2.065572	1.414308
PD	-1.355465	-2.038530	-1.010706
PD	-1.879182	-2.162950	1.696862

2.- More stable structures for the $Pd_{17}Cu_2$ nanocluster

Isomer a)

CU	1.537548	0.242733	0.000595
PD	0.179577	2.566583	0.008042
PD	0.183642	0.952519	-2.311264
PD	0.241986	-1.625003	-1.490102
PD	0.247168	-1.632062	1.483865
PD	0.187116	0.939955	2.317607
CU	-0.941782	0.189574	0.001297
PD	-3.552054	0.063102	0.000195
PD	-2.098349	-0.529208	2.310678
PD	-2.136213	2.066416	1.380345
PD	-2.135444	2.080014	-1.365834
PD	-2.100107	-0.511064	-2.314452
PD	-1.963115	-2.206686	-0.003649
PD	2.526529	-0.399193	-2.291000
PD	2.432161	-2.185251	-0.011052
PD	2.531427	-0.415048	2.285627
PD	2.433398	2.201036	1.427172
PD	2.427248	2.212065	-1.419198
PD	0.239284	-3.836314	-0.008109

Isomer b)

CU	1.202482	-0.002098	0.008915
PD	2.323306	2.271848	-0.735541
PD	2.302101	1.370770	1.921624
PD	2.296431	-1.394321	1.909446
PD	2.323002	-2.268069	-0.747961
PD	2.286985	0.005203	-2.340934
PD	-2.287310	0.010747	-2.341347
PD	-2.313568	2.279678	-0.731605
PD	-2.297648	1.374934	1.920849
PD	-2.302567	-1.388166	1.906520
PD	-2.330729	-2.263898	-0.753712
PD	-3.821534	0.009963	-0.013700
CU	-1.202559	0.001732	0.009992
PD	-0.003818	-2.271340	0.693647
PD	-0.001244	-0.014586	2.405119
PD	0.004184	2.262241	0.716285
PD	0.002053	1.435074	-1.898205
PD	-0.000891	-1.427289	-1.906933
PD	3.821291	0.007431	-0.014843

Isomer c)

CU	1.558545	0.193077	-0.001354
PD	0.272566	2.566483	0.061844
PD	0.234489	1.001804	-2.277772
PD	0.187963	-1.596443	-1.525609
PD	0.187795	-1.668499	1.444644
PD	0.226627	0.854925	2.313891
CU	-0.927618	0.227043	-0.000318
PD	-3.542064	0.191344	-0.000841
PD	-2.133434	-0.538853	2.292704
PD	-2.053249	2.112491	1.418290
PD	-2.069097	2.178592	-1.320360
PD	-2.134285	-0.385764	-2.306458
PD	-2.059273	-2.129462	-0.053408
PD	2.503361	-0.452786	-2.316757
PD	2.370280	-2.234019	-0.062073
PD	2.508527	-0.577109	2.285418
PD	2.524127	2.082447	1.458517
PD	2.533794	2.156663	-1.349114
PD	0.065131	-3.812678	-0.061918

Isomer d)

CU	1.200144	0.000566	0.000734
PD	2.310192	0.936428	-2.161194
PD	2.290451	2.362654	0.218995
PD	2.306548	0.520610	2.298684
PD	2.330776	-2.009743	1.187346
PD	2.328308	-1.755347	-1.538022
PD	-2.325863	-1.757614	-1.539471
PD	-2.309740	0.936238	-2.161411
PD	-2.292753	2.360048	0.219002
PD	-2.308209	0.520739	2.296397
PD	-2.329729	-2.011232	1.186999
PD	-3.862886	-0.011882	-0.000737
CU	-1.200032	-0.000484	-0.000022
PD	-0.000275	-0.962320	2.181480
PD	-0.000349	1.778322	1.579162
PD	-0.000409	2.041421	-1.224966
PD	0.000487	-0.539008	-2.321018
PD	0.000814	-2.397499	-0.220751
PD	3.862569	-0.011863	-0.000919

Isomer e)

CU	1.216657	0.003192	-0.000796
PD	2.341987	1.873499	-1.470476
PD	2.346543	1.963451	1.346270
PD	2.318690	-0.656723	2.249745
PD	2.338832	-2.395464	0.075297
PD	2.318856	-0.803038	-2.204665
PD	-2.321483	-0.802811	-2.204733
PD	-2.343426	1.874167	-1.468037
PD	-2.345001	1.963298	1.348361
PD	-2.318020	-0.659279	2.250623
PD	-2.336515	-2.396167	0.076119
PD	-3.825057	-0.012376	0.001515
CU	-1.216894	0.003761	-0.000028
PD	0.000810	-1.882913	1.424336
PD	0.000622	0.857609	2.219569
PD	-0.000380	2.343980	-0.073031
PD	-0.002166	0.712452	-2.269599
PD	0.000019	-1.971881	-1.301234
PD	3.825830	-0.011956	0.000432

Isomer f)

CU	1.527661	0.262135	-0.000272
PD	0.114972	2.552369	0.035781
PD	0.150733	1.008237	-2.288026
PD	0.276070	-1.578887	-1.523819
PD	0.296000	-1.637047	1.450966
PD	0.161115	0.909907	2.325857
CU	-0.952099	0.172306	0.000939
PD	-3.550661	-0.003138	0.002074
PD	-2.098012	-0.629096	2.297618
PD	-2.214461	1.981344	1.416787
PD	-2.224975	2.068606	-1.341036
PD	-2.110565	-0.513654	-2.306931
PD	-1.914597	-2.249564	-0.014956
PD	2.559855	-0.297115	-2.289227
PD	2.491746	-2.148599	-0.047977
PD	2.568942	-0.400468	2.272795
PD	2.412664	2.217243	1.448219
PD	2.410706	2.280375	-1.363355
PD	0.326785	-3.819928	-0.075169

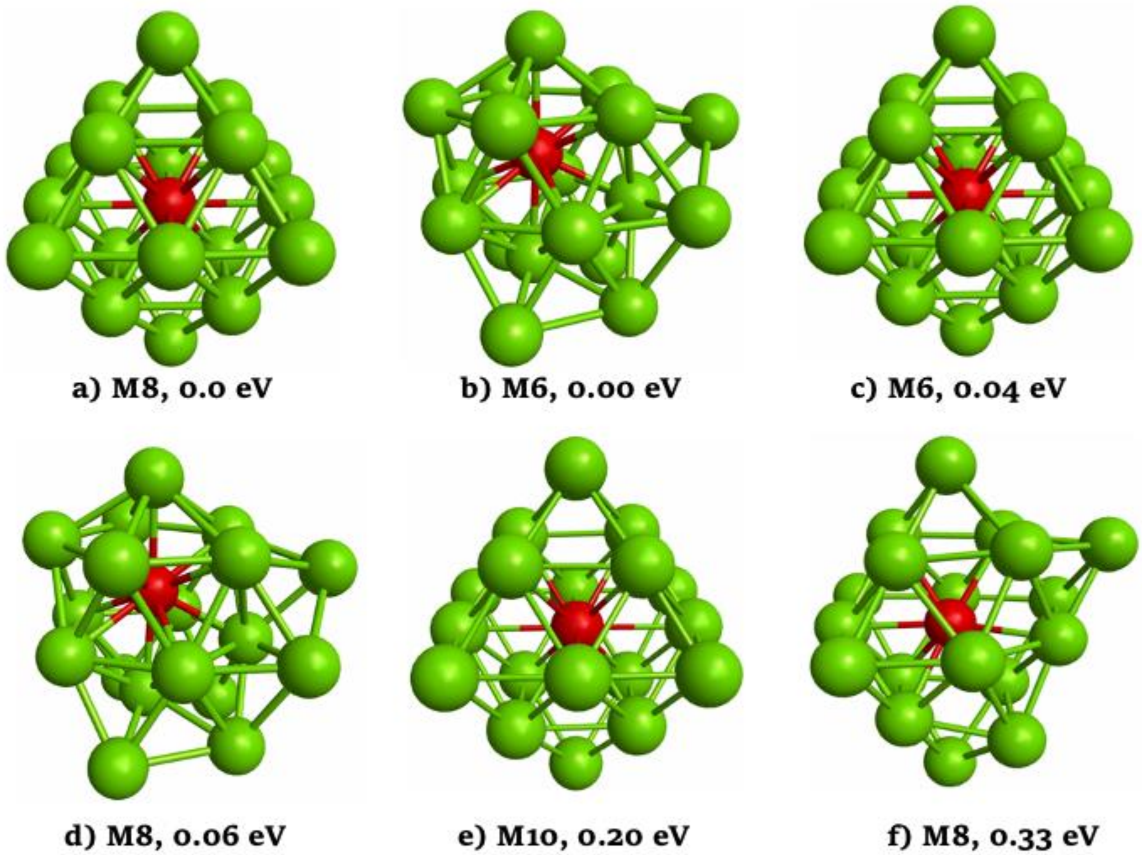

Figure SI-1. The most stable structures of the $Pd_{18}Cu$ nanocluster. Green and red spheres represent Pd and Cu atoms, respectively. The corresponding spin multiplicity (M) and the relative stability energy (in eV) of each cluster isomer with respect to the lowest energy structure are given. The relative stabilities were obtained by performing single point calculations employing the meta-GGA TPSS functional in combination with the QECP18-SD basis set for the Pd atoms and the TZVP-GGA basis set for the Cu atoms.

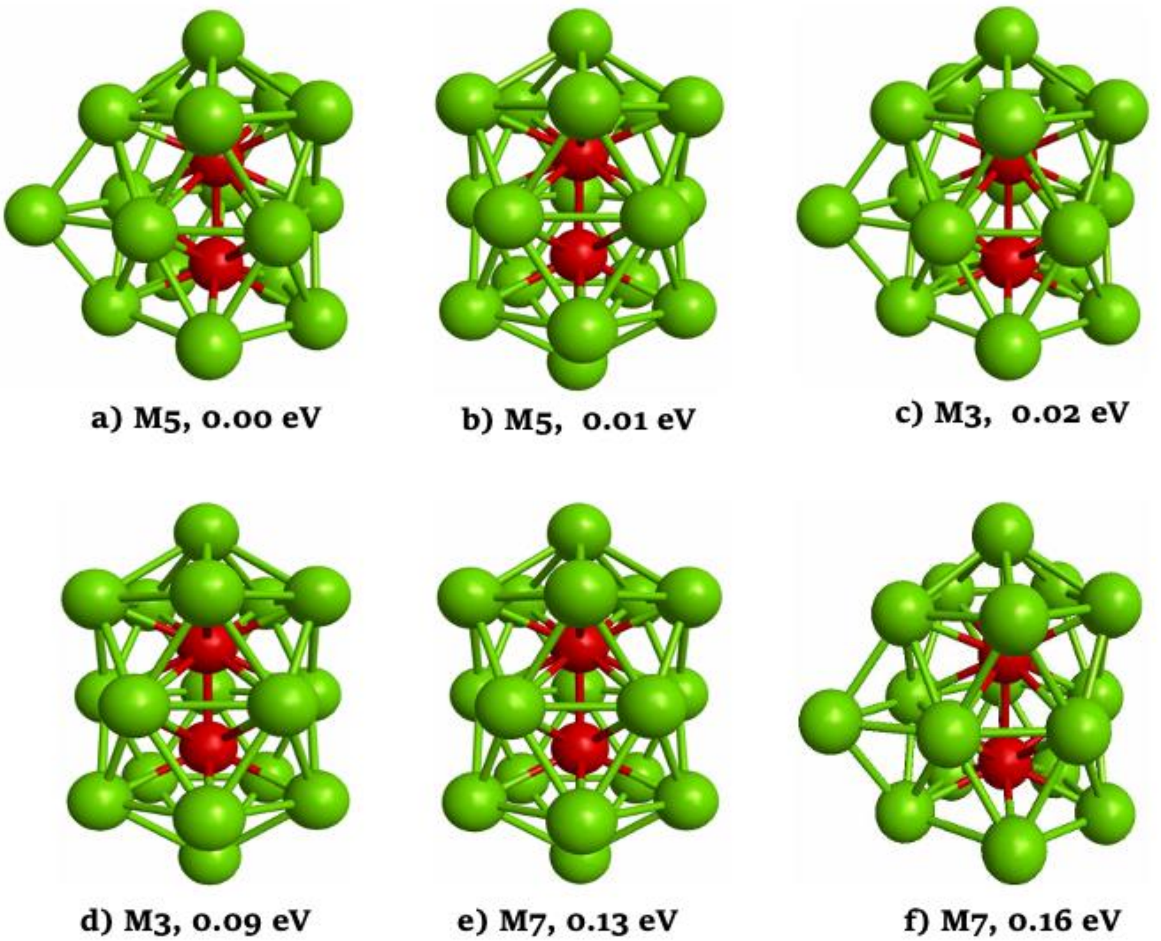

Figure SI-2. The most stable structures of the $Pd_{17}Cu_2$ nanocluster. Green and red spheres represent Pd and Cu atoms, respectively. The corresponding spin multiplicity (M) and the relative stability energy (in eV) of each cluster isomer with respect to the lowest energy structure are given. The relative stabilities were obtained by performing single point calculations employing the meta-GGA TPSS functional in combination with the QECP18-SD basis set for the Pd atoms and the TZVP-GGA basis set for the Cu atoms.
